# Comparison of Psychometric Functions Measured Using Remote Testing and Laboratory Testing †

**DOI:** 10.3390/audiolres14030039

**Published:** 2024-05-22

**Authors:** Nirmal Srinivasan, Chhayakanta Patro, Radhika Kansangra, Angelica Trotman

**Affiliations:** Department of Speech-Language Pathology and Audiology, Towson University, Towson, MD 21252, USA; cpatro@towson.edu (C.P.); rkansa2@students.towson.edu (R.K.); atrotm3@students.towson.edu (A.T.)

**Keywords:** remote testing, laboratory testing, psychometric functions, speech intelligibility

## Abstract

The use of remote testing to collect behavioral data has been on the rise, especially after the COVID-19 pandemic. Here we present psychometric functions for a commonly used speech corpus obtained in remote testing and laboratory testing conditions on young normal hearing listeners in the presence of different types of maskers. Headphone use for the remote testing group was checked by supplementing procedures from prior literature using a Huggins pitch task. Results revealed no significant differences in the measured thresholds using the remote testing and laboratory testing conditions for all the three masker types. Also, the thresholds measured obtained in these two conditions were strongly correlated for a different group of young normal hearing listeners. Based on the results, excellent outcomes on auditory threshold measurements where the stimuli are presented both at levels lower than and above an individual’s speech-recognition threshold can be obtained by remotely testing the listeners.

## 1. Introduction

Evaluating an individual’s hearing ability reliably and accurately is very important for clinicians and researcher alike [1,2,3]. The implementation of strict social distancing protocols during the COVID-19 pandemic has underscored the challenges in reliably conducting in-person tests for measuring hearing acuity. However, this had opened up a new avenue for remote testing and measuring psychophysical thresholds. Accurately measuring psychoacoustic thresholds outside the confines of specialized laboratories could initiate important changes in how data is collected and would increase accessibility for basic and translational research [4]. Previous studies have shown success in comparing equivalent data collected in the lab versus remotely with acoustic hearing [5,6,7,8,9] as well as with electric hearing [10,11,12,13]. For instance, Lelo de Larrea-Mancera et al. (2022) [5] measured performance on a battery of psychoacoustic experiments in tasks involving audibility, temporal fine structure sensitivity, spectro-temproal modulation sensitivity, and spatial release from masking from a large group of young normal-hearing listeners. The results indicated that the suprathreshold auditory processing thresholds obtained using uncalibrated, participant-owned devices in remote settings were comparable to the thresholds obtained in the laboratory with calibrated devices. Similarly, Mok et al. (2021) [7] compared web-based measurements to lab-based thresholds on a range of classic psychoacoustic tasks such as fundamental frequency discrimination, gap detection, and sensitivity to interaural time and level differences, co-modulation masking release, word identification, and consonant confusion. The results revealed an excellent agreement between the thresholds collected using web-based and lab-based measurements indicating robustness in thresholds. Soares et al., (2021) [14] compared thresholds from psychoacoustic tasks that included frequency discrimination, amplitude modulation detection, binaural hearing, and temporal gap detection using a mobile system and a laboratory-based system and reported no differences in thresholds between the two methods. De Graff et al. (2018) [10] assessed speech recognition abilities of cochlear implant users when the test was self-administered or administered by a clinician and found no significant differences in the thresholds between the two modes of administration. 

In many of the web-based studies that measured various auditory processing thresholds, the target stimuli were often presented at levels higher than an individual’s speech recognition threshold. However, while measuring a psychometric function, it is essential to present the target stimuli at levels lower than and above an individual’s speech-recognition thresholds to obtain a comprehensive understanding of the function. The extent to which the behavioral thresholds may differ at these presentation levels remains unclear. This experiment aimed to measure the psychometric function for a commonly used speech corpus using both remote testing and laboratory testing conditions in two distinct groups of listeners. Additionally, psychometric functions measured for the same group of listeners under remote and laboratory testing conditions are also reported. The primary objective was to examine the differences in the psychometric functions based on the method of acquisition. Another objective was to investigate whether the measured psychometric functions differ with masker types and the positioning of the masker relative to the target. 

## 2. Materials and Methods

### 2.1. Participants

Twenty-five young listeners (mean age = 22.8 years old, range = 19–27 years old) participated in the remote testing group and a different set of 25 young listeners (mean age = 22.4 years old, range = 19–26 years old) participated in the laboratory testing group of the study. All participants in the remote testing group completed a speech and hearing screening as a requirement of the Speech-Language Pathology and Audiology undergraduate program at Towson University and all of them reported normal hearing. All the listeners in the laboratory testing group had normal hearing (defined as ≤15 dB HL) at all octave frequencies between 250 and 8000 Hz and no asymmetry (>10 dB) between the ears at all tested frequencies. A smaller subset of ten young listeners (mean age = 22.1 years old, range = 20–23 years old) completed both the remote testing and the laboratory testing. This third group also had normal hearing as defined earlier for the laboratory testing group. One half of the third group of listeners completed remote testing first followed by laboratory testing while the other half completed laboratory testing followed by remote testing. All testing procedures were approved by the Institutional Review Board at Towson University. All the participants were monetarily compensated for their time.

### 2.2. Stimuli

Three male talkers from the Coordinate Response Measure (CRM) [15] were used for the target sentences. All sentences in the CRM corpus have the form “Ready [CALL SIGN] go to [COLOR] [NUMBER] now”. There are eight possible call signs (Arrow, Baron, Charlie, Eagle, Hopper, Laker, Ringo, and Tiger), four colors (Blue, Red, White, and Green) and eight numbers (1–8). All possible combinations of the call signs, colors, and numbers were used. Three of the four male talkers were used as the target talker. The fourth talker in the corpus had a slower speaking rate than the other three talkers and hence was not used. 

The maskers were either different CRM sentences (speech masker) or Gaussian noise (noise masker). In the speech masker condition, the stimuli consisted of three simultaneous phrases from the CRM corpus: a target phrase with the call sign “Charlie” and two masker phrases with randomly selected call signs other than “Charlie”. The target and the maskers would come either from the same location in front of the listener (colocated condition) or the target coming directly in front of the listener and the maskers were symmetrically spatially separated by ±45° (separated condition). The target talker and the masker talkers varied from trial to trial. In each trial, the target and maskers were selected randomly from the corpus such that different colors and different numbers were used for the three phrases. In the noise masker condition, the masking noise was spectrally shaped with a 512-point FIR filter to match the average overall spectrum of the 756 CRM sentences spoken by the three target talkers and was rectangularly gated to the length of the target phrase. The overall level of the target was adjusted relatively to the maskers to produce one of 12 signal-to-noise ratios (SNRs) ranging from −18 dB to 15 dB in 3 dB steps. Each of the SNRs were presented 25 times resulting in a total of 300 trials per condition.

For the speech masker condition, virtual acoustic techniques were used to simulate an anechoic room (dimensions: 5.7 m (length) × 4.3 m (width) × 2.6 m (height)) using head-related impulse responses (HRIRs). Simulation techniques as described in Zahorik (2009) [16] were used to generate HRIRs. Briefly, an image model was used to compute directions, delays, and attenuations, which were then spatially rendered along with the direct path using non-individualized head-related transfer functions. Within the simulated anechoic space, the listener was positioned in the center of the space and the speech source was positioned 1.4 m in front of the listener. Two spatial configurations were used: colocated (all three sentences presented from 0° azimuth) and spatially separated condition (target at 0°, symmetrical maskers at ±45°). Target and masking speech were convolved with the HRIRs for their appropriate locations relative to the listener and were presented binaurally over headphones. 

### 2.3. Procedure

#### 2.3.1. Remote Testing

The MATLAB compiler was used to create an executable file (for Windows operating system) that contained all the required MATLAB files, HRIRs, CRM audio files, and installation instructions to run the required experiments. During the initial recruitment, the interested participants were asked to confirm whether they owned a device with the Windows operating system and whether their sound card was working. Participants without a Microsoft Windows based computer were excluded from the study. All the participants in the remote testing group used their personal headphones to complete the study. After electronically signing the informed consent forms, the participants were directed to a password protected SharePoint website to download the MATLAB executable file. They were also given an option to schedule a video conferencing session to troubleshoot any issues encountered while downloading or installing the executable. 

Following installation, participants were initially presented with a headphone screening test utilizing Huggins pitch to assess headphones use [17]. This task presented three intervals of 1000 ms white noise. Two of the intervals contained diotic presentation of the above-mentioned white noise while the third interval contained the Huggins pitch (HP) stimulus in which one ear was presented with a white noise stimulus while the other ear was presented with the same white noise but with a phase shift of 180° over a narrow frequency band [18,19]. A center frequency of 600 Hz was used (roughly in the middle of the frequency region where HP was salient) and white noise was created by generating a random sequence of Gaussian distributed numbers with a mean of 0. The HP signals were generated by introducing a constant phase shift of 180° in a frequency band of 600 Hz (±6%). The phase shifted version was presented to the right ear while the original version of the noise was delivered to the left ear [20]. 

During the headphone check task, the participants were informed that “they will hear three noise bursts with silent gaps in between and one of the three bursts will contain a hidden beep”. A three-alternative forced-choice technique was used to present the signals and the participants were instructed to pick the interval that contained the hidden beep. Overall, 10 trials were presented, and six trials were randomly selected without replacement for analysis. The participants were assumed to use their headphones if they had correctly identified the interval that elicited the HP percept in all the six randomly picked trials. If the participants failed the headphone check task, they were re-instructed and would complete the task one more time. All 25 participants passed their headphone check task during their first attempt. It should be noted that this test checks whether the participants are using headphones to complete the experimental tasks and does not check for headphone quality or functionality.

After passing the headphone check task, the participants completed various test conditions at a pre-determined random order generated by the software. The participants were instructed to set the volume level of their computer to a comfortable listening level and were asked not to change the volume level during the various conditions. During every trial, responses were obtained via a custom MATLAB GUI having a grid of four colors and eight numbers and feedback was provided after each trial in the form of “correct” or “incorrect”. Data collection was self-paced, and the participants were instructed to take breaks whenever they needed to. After the completion of all test conditions, the participants were instructed to upload the output data files to a different password protected SharePoint website. 

#### 2.3.2. Laboratory Testing

The listeners were seated in a sound treated booth in the Speech-Language Pathology and Audiology Department at Towson University and listened to the auditory stimuli presented over circumaural headphones (Sennheiser HD 650, Sennheiser, Hanover, Germany). The stimuli were generated using MATLAB on the lab computer and delivered through a Lynx Hilo sound card. The auditory stimuli were presented to the listeners at a 20 dB sensation level (SL) relative to the listener’s four-frequency pure tone average (the average of audiometric thresholds for the octave frequencies 0.5, 1, 2, and 4 kHz) and was kept constant during the experiment. Responses were recorded using a touchscreen monitor located in the test booth directly in front of the listener. Feedback in the form of “correct” or “incorrect” was displayed on the monitor following each trial. Data collection was self-paced, and the participants were instructed to take breaks whenever they needed.

#### 2.3.3. Data Analysis

Analyses were performed with SPSS 28.0 (IBM Corp., Armonk, NY, USA). Three mixed ANOVAs were used to investigate the differences between the remote testing and laboratory testing conditions for the three different kinds of maskers. Pearson’s correlations were used to test the test-retest repeatability of the thresholds obtained. 

## 3. Results

**Figure 1:**
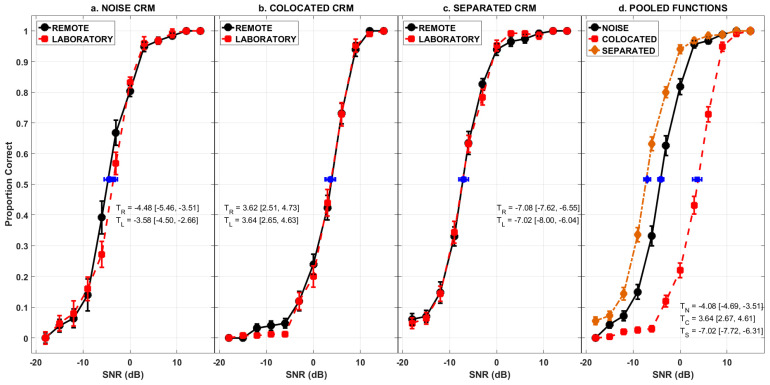
Panel (**a**–**c**) shows the psychometric functions obtained using the remote testing (black circles, solid line) and laboratory testing (red squares, broken line) conditions for the noise masker (panel (**a**)), colocated speech masker (panel (**b**)), and spatially separated speech masker (panel (**c**)). The blue circle and the blue square show the threshold (51.56% point on the psychometric function) for the remote testing and the laboratory testing conditions. The thresholds along with the 95% confidence intervals are shown as well (subscript R indicates remote threshold and subscript L indicates laboratory testing). Panel (**d**) shows the pooled psychometric functions for the noise masker (black circles, solid line), colocated speech masker (red square, broken line) and spatially separated speech masker (brown diamond, dash-dot line). The blue circle, the blue square and the blue diamond show the threshold (51.56% point on the psychometric function) for the noise, colocated, and spatially separated speech maskers respectively. The thresholds along with the 95% confidence intervals are shown as well (subscript N indicates noise masker, subscript C indicates colocated speech masker and subscript S indicates spatially separated speech masker thresholds). The error bars for the proportion correct data in all the panels indicate ± 1 SEM.

Figure 1a–c, show the percentage of correctly identified key words at the various SNRs in both the remote testing and the laboratory testing conditions, for the three different masking noise conditions. Logistic functions were fit to the averaged data pooled across all the listeners in the remote testing and the laboratory testing conditions using a maximum-likelihood algorithm to approximate the psychometric functions which are shown in solid lines in Figure 1. The SNR corresponding to the midpoint between perfect performance (100%) and chance performance (3.125%), which is 51.56% is also potted along with the 95% confidence intervals for the mid-point of the fitted psychometric functions which were estimated using a bootstrapping procedure [21,22,23].

A mixed factorial ANOVA was conducted on the percentage of key words correctly identified in the presence of various maskers as the dependent variable with testing condition (remote testing and laboratory testing) as the between-subject factors and different SNRs. 

(−18 dB to 15 dB in 3 dB steps) as the within-subject factor. Results indicated no significant main effect of testing condition on key word recognition (Noise Masker: *F*(1,48) = 0.778, *p* = 0.382, *partial η*^2^ = 0.016; Colocated Masker: *F* (1,48) = 0.719, *p* = 0.401, *partial η*^2^ = 0.015; Separated Masker: *F* (1,48) = 0.001, *p* = 0.976, *partial η*^2^ = 0.001) and no significant interaction between testing condition and SNR on key word recognition (Noise Masker: *F* (11,528) = 1.526, *p* = 0.118, *partial η*^2^ = 0.031; Colocated Masker: *F* (11,528) = 0.385, *p* = 0.962, *partial η*^2^ = 0.008; Separated Masker: *F* (11,528) = 0.438, *p* = 0.939, *partial η*^2^ = 0.009). Table 1 shows the results of these mixed factorial ANOVAs as well.

Psychometric functions, as explained earlier, for the individual listeners were computed as well. The mid-point (51.56%) of the psychometric function, denoted as threshold from hereon, and the slope of the individual psychometric function for listeners were used as the dependent variable to see if they differed between the remote testing and the laboratory testing conditions for the noise, the colocated and separated maskers. Independent group t-tests revealed no significant differences in the thresholds (Noise Masker: *t* (48) = −1.622, *p* = 0.111, *Cohen’s d* = −0.459, 95% CI [−1.108, 0.105]; Colocated Masker: *t* (48) = −0.124, *p* = 0.902, *Cohen’s d* = −0.035, 95% CI [−0.589, 0.519]; Separated Masker: *t* (48) = 0.349, *p* = 0.729, *Cohen’s d* = 0.099, 95% CI [−0.456, 0.653]) and the slope of the psychometric functions (Noise Masker: *t* (48) = 1.884, *p* = 0.066, *Cohen’s d* = 0.533, 95% CI [−0.034, 1.095]; Colocated Masker: *t* (48) = 1.314, *p* = 0.195, *Cohen’s d* = 0.372, 95% CI [−0.190, 0.929]; Separated Masker: *t* (48) = 1.190, *p* = 0.240, *Cohen’s d* = 0.377, 95% CI [−0.223, 0.893]) between the remote testing and the laboratory testing conditions for all the three masker types.

A mixed factorial ANOVA was conducted on the individual psychometric function thresholds with testing condition (remote testing and laboratory testing) as the between-subject factors and spatial location of the masker (colocated vs separated) as the within-subject factor. The results indicated a significant main effect of the spatial location of the masker (*F* (1,48) = 698.232, *p* < 0.001, *partial η*^2^ = 0.936) with the spatially separated thresholds being significantly better than the colocated thresholds. There was no significant main effect of the testing condition (*F* (1,48) = 0.032, *p* = 0.858, *partial η*^2^ = 0.001) or interaction between spatial location and the testing condition (*F* (1,48) = 0.122, *p* = 0.728, *partial η*^2^ = 0.003).

Since there was no significant difference in the percentage of key-words correctly identified between the remote testing and the laboratory testing conditions, the data were pooled, and the overall psychometric function is displayed in Figure 1, panel d. The mean thresholds and the 95% confidence intervals for the thresholds are also shown in Figure 1. One way ANOVA revealed a significant effect of the masker type on the slope of the psychometric functions (*F* (2,98) = 5.503, *p* = 0.005, *partial η*^2^ = 0.101). Post-hoc analysis with Bonferroni correction indicated that the slope of the psychometric function with the noise masker was significantly greater than the slopes of the functions with the colocated and separated speech maskers. 

**Figure 2:**
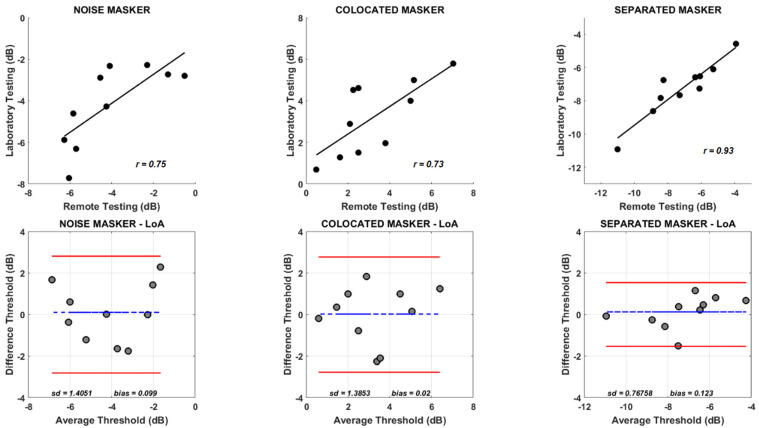
The top row shows the scatterplot of thresholds obtained using the remote testing and laboratory testing conditions for the noise masker (**left panel**), colocated speech masker (**middle panel**), and spatially separated speech masker (**right panel**). The solid black line inside the panel shows the best fit line for the data. Bold correlations inside the panels are significant at *p* < 0.05. The bottom row shows the mean difference and limits of agreement for thresholds estimated using the remote testing and laboratory testing conditions for the noise masker (**left panel**), colocated speech masker (**middle panel**), and spatially separated speech masker (**right panel**). The broken line in each panel in the bottom row indicates the mean difference between the two experimental conditions. The solid red lines indicate limits of agreement (mean difference ±1.96 × standard deviation of the mean difference).

The test-retest repeatability of the thresholds obtained was assessed by having a new set of 10 listeners complete both the remote testing and laboratory testing in randomized order. The top panel in Figure 2 shows the scatter plot of the thresholds obtained for all three types of maskers for these 10 listeners. Thresholds obtained using the two testing conditions for all the three maskers were positively correlated, indicating a low with-in subject variability and a high test-retest repeatability. Internal consistency between the thresholds using Cronbach’s alpha [24]. indicated high levels of reliability between the thresholds for all the three masker types (*α_Noise_* = 0.85; *α_Colocated_* = 0.84; *α_Separated_* = 0.90). The bottom panels in Figure 2 show the test-retest reliability using limits of agreement [23]. The broken blue lines in all bottom panels show the mean difference in thresholds obtained using the remote testing and the laboratory testing methods, and any deviation of this mean difference line from 0 indicates the presence of a measurement bias. The solid red lines indicate 95% limits of agreement (mean difference between the experimental conditions ±1.96 × standard deviation of the mean difference between the experimental conditions). As seen from Figure 2, most of the points fall within the limits of agreement. Also, the bias estimates for the three masker types were small (range: 0.02–0.12) suggesting similar threshold estimates were obtained when using the remote testing of the laboratory testing methods.

## 4. Discussion

The main focus of this study was to compare the psychometric functions and the thresholds obtained using remote testing and laboratory testing. The second goal was to compare the thresholds obtained using remote testing and laboratory testing in the same group of listeners to check the test-retest reliability. The resulting psychometric function for all the three kinds of maskers showed a good fit for a sigmoidal curve which is typical of most measures of speech intelligibility. Target identification in the presence of all the three maskers resulted in an increased percent correct performance as the SNR increased. No significant differences in the thresholds between the remote testing and the laboratory testing conditions for all the three types of maskers is consistent with the findings of Lelo de Larrea-Mancera et al. (2022) [5] and Soares et al. (2021) [14]. 

Furthermore, the thresholds obtained here are in close agreement with corresponding thresholds reported in the literature. The threshold obtained for the noise masker (*M* = −4.08 dB, 95% CI [−4.69, −3.47]) and the shape of the psychometric function were consistent with those reported by Brungart (2001) [25]. The threshold obtained for the colocated speech masker (*M* = 3.64 dB, 95% CI [2.67, 4.61]) was similar to the thresholds reported earlier in the literature [26,27,28,29]. The shape of the psychometric function was consistent with that reported earlier as well [26,27]. The speech identification thresholds obtained for the spatially separated speech masker (*M* = −7.02 dB, 95% CI [−7.72, −6.31]) were similar to the thresholds reported earlier in the literature [29,30]. Speech identification thresholds improved when the target was spatially separated from the masker which is in agreement with several other studies in the literature that used CRM [28,29,31,32,33,34] and other closed set speech corpora [35]. 

The mean difference between the thresholds obtained from the remote testing and the laboratory testing from a different group of listeners who performed both tasks in a random order was close to zero for all of the three masker conditions, indicating little systematic bias. This suggests that there were similar measurement errors in both sessions and that there was little to no bias in estimating the thresholds using either of the methods. This analysis demonstrated the range of alignment to be expected between different threshold estimates within subjects and indicates that either of the conditions (remote testing or laboratory testing) produced minimally biased estimates at the group level.

Even though the effect sizes (*Cohen’s d*) were moderate, there was no significant difference (*p* > 0.05) in the mid-point of the psychometric function and the slope of the psychometric function between the remote testing and the laboratory testing for the noise masker. This indicates that the study might be underpowered to detect these subtle relationships between speech understanding and SNR in the noise masker condition. One probable cause could be the lack of control over the listening environment in the remote testing condition.

Another issue of potential concern was the presentation level of the stimulus in the testing conditions. Jakien et al. (2017) [32] concluded that speech identification thresholds improved with an increase in overall presentation levels, and this improvement was small relative to the improvement because of the spatial separation between the target and the maskers. Oh et al. (2023) [36] concluded that the amount of spatial release from masking (defined as the improvement in speech identification thresholds because of spatially separating the maskers from the target) was significantly affected by presentation levels and those improvements are noticeable for smaller separations (<15°) between the target and the masker. We did not collect presentation level or system volume level data on the group of listeners who performed the tasks in remote conditions. For the smaller group that completed both conditions, auditory stimuli were presented at 20 dB SL in the laboratory testing condition. They were instructed to pick a comfortable listening level for the remote testing condition. When their comfortable listening level was measured using a sound level meter, the average listening level was around 35 dB SL (range: 20–42 dB SL). The average SL measurements indicate that presentation level has little to no effect on the measured psychometric functions using remote testing and laboratory testing methods.

Overall, the results presented here indicate that psychometric functions can be obtained remotely and measured reliably using people’s own devices in their home environments. The consistency (both between and within groups) of the results using both data collection techniques indicate that reliable auditory measurements can be obtained by remotely testing in young normal hearing listeners.

## Figures and Tables

**Table 1:** Results of the mixed factorial ANOVAS with proportion keywords correctly identified in the presence of various maskers as the dependent variable with testing condition (remote testing and laboratory testing) as the between-subject factors and different SNRs (−18 dB to 15 dB in 3 dB steps) as the within-subject factor for noise, colocated, and separated maskers.

Noise Masker					
*Source*	*df*	*MS*	*F*	*p*	*partial η* ^2^
Condition (A)	(1,48)	0.014	0.778	0.382	0.016
TMR (B)	(11,528)	9.082	555.072	< 0.001	0.92
A × B	(11,528)	0.025	1.526	0.118	0.031
**Colocated Masker**					
***Source***	***df***	***MS***	***F***	***p***	***partial η* ^2^**
Condition (A)	(1,48)	0.014	0.719	0.401	0.015
TMR (B)	(11,528)	8.905	770.655	<0.001	0.941
A × B	(11,528)	0.004	0.385	0.962	0.008
**Separated Masker**					
***Source***	***df***	***MS***	***F***	***p***	***partial η* ^2^**
Condition (A)	(1,48)	0.014	0.001	0.976	0.000
TMR (B)	(11,528)	7.844	784.438	<0.001	0.942
A × B	(11,528)	0.004	0.438	0.942	0.009

*Note*. MS indicates mean sum of squares due to the source.

## Data Availability

The data that support the findings of this study are available from the corresponding author, Nirmal Srinivasan, upon reasonable request.
